# Introduction of the Kaifu telemedicine system for emergency medicine to ambulance services with improvement of the survival rates

**DOI:** 10.1186/cc13258

**Published:** 2014-03-17

**Authors:** F Obata, R Tabata, K Mori, T Kageji, K Tani, H Bando

**Affiliations:** 1 Tokushima Prefectural Kaifu Hospital, Tokushima, Japan; 2 Institute of Health Biosciences, University of Tokushima Graduate School, Tokushima, Japan; 3 Tokushima University Hospital, Tokushima, Japan

## Introduction

Our hospital has introduced the telemedicine system for emergency medicine (k-support) using a smart device to rectify health disparity and reduce the burden to general physicians since February 2013. We have expanded to the ambulance services' k-support toward further survival rate improvement.

## Methods

The registration device, patient information and image information such as magnetic resonance imaging and computed tomography taken in the hospital were transferred in real time to k-support using the smart device. Since September 2013, we newly developed k-support to the Kainan Fire Department Mugi branch office, Kainan Fire Department Hiwasa branch office, Kaifu Fire-fighting Union Kainan fire department, and Muroto Fire-Fighting Headquarters Touyou branch office, and reviewed our experience of this approach in the use of k-support during the first 3 months of the introduction.

## Results

k-support was used for 62 patients, and 13 (21.0%) of them were categorized as neurosurgical diseases. The detail of neurosurgical diseases consisted of six patients (46.1%) with head injury, followed by five patients (38.5%) with cerebral infarction, one patient (7.7%) with cerebral hemorrhage and one patient (7.7%) with miscellaneous diseases. We shared the information by ambulance services to tweet the patient information or transmit vital signs and electrocardiograms. We experienced a case that was diagnosed with cardiogenic embolism and tried the 'drip and ship' method of rt-PA. The outcomes were as follows: hospitalization, 28 (45.2%); discharge, 18 (29.0%); and transfer, 16 (25.8%).

## Conclusion

Introduction of k-support is the first trial in Japan. For emergency diseases such as ischemic heart diseases and stroke, we could perform responses earlier than ever by providing this system.

